# Editorial: Bioprospecting and Biotechnology of Extremophiles

**DOI:** 10.3389/fbioe.2019.00204

**Published:** 2019-08-23

**Authors:** Milko A. Jorquera, Steffen P. Graether, Fumito Maruyama

**Affiliations:** ^1^Laboratorio de Ecología Microbiana Aplicada, Departamento de Ciencias Químicas y Recursos Naturales, Universidad de La Frontera, Temuco, Chile; ^2^Network for Extreme Environment Research, Scientific and Technological Bioresource Nucleus, Universidad de La Frontera, Temuco, Chile; ^3^Department of Molecular and Cellular Biology, University of Guelph, Guelph, ON, Canada; ^4^Office of Industry-Academia-Government and Community Collaboration, Hiroshima University, Hiroshima, Japan

**Keywords:** airborne, andean highland, bacteria, fish, hydrothermal vent, hot spring, microorganisms, plants

Extreme environments are defined as those environments that contains conditions that are difficult to survive for most life forms. They include natural environments (hyper-arid deserts and rocks, oceanic deeps, salt lakes, volcanoes, high mountains, and upper atmosphere, etc.), as well as artificial ones (operating rooms, aircrafts, spaceships, etc.). Because most of the earth's surface (>80%) is covered by extreme environments, it is not surprising that a wide diversity of organisms (e.g., animals, plants, insects, fungi, and bacteria) are able to colonize, survive, and proliferate in these extreme environments. Those organisms living in extreme environments, also known as extremophiles, have evolved and developed a wide variety of strategies and mechanisms to live under harsh conditions, such as exceptionally high or low values of temperature, pressure, oxygen, carbon dioxide, acidity, radiation, nutrient content, water content, salt content, sugars, etc. Thus, extremophiles have opened a new window into bioprospecting for the discovery and application of novel bioactive products.

In this sense, the classic example of extremophiles and their bioactive compounds is the DNA polymerase from thermophilic bacteria (e.g., *Thermus aquaticus*) from hydrothermal environments (Chien et al., 1976). Without a doubt, the advances in molecular biology and life sciences during the last three decades would not have been possible without these extremozymes. Another example is the cold-active enzymes from Artic or Antarctic extremophiles (animals, plants, and microorganisms), which are highly attractive for biotechnologists since they can be used in food processing to improve milk fermentation, to store frozen yogurt, and to improve ice-cream production (Cid et al., 2016). Therefore, extremozymes and bioactive compounds produced by extremophiles, and their potential application in biotechnology, have been recognized in various fields, such as medicine (e.g., antibiotics and antitumors), food technology (e.g., phytases and phosphatases), biofuel production (e.g., proteases and lipases), cosmetic industry (carotenoids), biomining and contaminated soil remediation (xenobiotic-degrading enzymes), agriculture (plant-growth inducers), and organic residue cycling (cellulose and lignocellulose) (Rampelotto, 2016; Dumorné et al., 2017).

This Research Topic highlights microbial communities inhabiting extreme environments or in extremophile microbiomes as an unexplored source of novel biological resources, with a huge potential for biotechnology and its industry. In this sense, ureolytic bacteria from desert salt lakes could be used as a new technology for bio-precipitation of ion crystals (e.g., calcium and magnesium), improving water use in mining. Similarly, the core microbiome of Arctic fish is a possible tool in aquatic biotechnology to assist in improving fish health and waste management practices in aquaculture. The bioprospecting of airborne microbial communities can give clues to their application in the degradation of toxic compounds and increased tolerance of heavy metal stress in plants. In a similar context, epoxide hydrolases found in the metagenomes from hot springs have also been proposed for use in the detoxication of xenobiotics, and as a defense against pathogen attack and stress response in plants.

In addition, climate change is affecting the distribution and abundance of flora and fauna in diverse regions around the globe, including extreme environments. The characterization of nitrous oxide (a recognized greenhouse gas) reducing bacteria in deep-sea hydrothermal vents is also noted in this Topic Research, emphasizing their capability to reduce gas emissions in the environment. With respect to climate change, microbial communities and plants from extreme environments are also being evaluated as biotechnological tools to improve the tolerance of cereal crops and trees from adverse climate events (such as droughts, flooding, frosts, hot, and cold waves, etc.), and can therefore prevent economical losses in agriculture. Plant-growth microorganisms from cold and hot deserts are possible alternatives to improve the salt tolerance of agriculturally relevant plants. Similarly, cold-stress related dehydrin genes can be a suitable tool in genetic engineering to improve cold tolerance in plants.

As noted in this Research Topic, advances in omics technologies have allowed for the investigation of extremophile properties and their bioactive compounds as has never been seen before, revealing their relevance in multiple disciplines in science, and in many aspects of bioprospecting and biotechnology, including medicine, bioenergy and biofuels, bioinformatics, biomaterials, biosafety and biosecurity, aquatic biotechnology, agriculture, environmental science, nanobiotechnology, industrial process, synthetic biology, DNA/protein engineering, and even more.

Lastly, we list here several international initiatives that could be useful for readers interested in extremophiles bioprospecting, such as: The Network for Extreme Environment Research (NEXER; http://www.nexerchile.cl/), The International Society for Extremophiles (https://www.extremophiles2018.org/), Swissaustral Chile Ltda. (http://www.swissaustral.ch/), Latin American Network of Extremophiles (https://redlae.science/), Spanish Network of Extremophiles Microorganisms (https://web.ua.es/en/rnme/spanish-network-of-extremophile-microorganisms.html), and the following related Research Topics in Frontiers:

1. Microbial Life Under Stress: Biochemical, Genomic, Transcriptomic, Proteomic, Bioinformatics, Evolutionary Aspects and Biotechnological Applications of Poly-Extremophilic Bacteria (https://www.frontiersin.org/research-topics/7007/microbial-life-under-stress-biochemical-genomic-transcriptomic-proteomic-bioinformatics-evolutionary).
2. Enzymes from Extreme Environments (https://www.frontiersin.org/research-topics/3054/enzymes-from-extreme-environments).
3. Extremophilic Industrially Important Enzymes and Molecular Mechanisms (https://www.frontiersin.org/research-topics/3700/extremophilic-industrially-important-enzymes-and-molecular-mechanisms).
4. Recent Advances in Acidophile Microbiology: Fundamentals and Applications (https://www.frontiersin.org/research-topics/5002/recent-advances-in-acidophile-microbiology-fundamentals-and-applications).
5. Actinobacteria, a Source of Biocatalytic Tools (https://www.frontiersin.org/research-topics/5513/actinobacteria-a-source-of-biocatalytic-tools).

## Author Contributions

MJ conceived of the idea for the Research Topic: Bioprospecting and Biotechnology of Extremophiles and served as editor. SG and FM served as co-editors for the Research Topic. MJ wrote the editorial, with editing help from SG and FM.

### Conflict of Interest Statement

The authors declare that the research was conducted in the absence of any commercial or financial relationships that could be construed as a potential conflict of interest.
